# Operations for Cataract

**Published:** 1858-12

**Authors:** R. Seeds

**Affiliations:** Portland


*Operations for Cataract. On the comparative merits and success of the principal operations performed upon the Human Eye for the removal of true Cataract from the axis of vision.* By R. SEEDS, M.D., Portland.

I shall commence with the process called solution or absorption. This is only applicable to soft cataracts and in these cases, I believe there is only one opinion among opthalmic surgeons upon the subject, which is, that it is the safest process to the eye; but the least satisfactory to the operator and the patient. A great discrepancy of opinion has existed among opthalmic surgeons with regard to the superiority of depression or couching, and its modification, Reclination, over Extraction. I shall now in a brief manner state the arguments on both sides of the question, commencing with Extraction.

The merits claimed for this operation are, the cataract being hard and not susceptible of absorption, it is removed from the eye at once, the incision of the cornea is healed in 24 or 48 hours; and that the opaque lens is removed in a few minutes, and sight restored immediately. These are the principal arguments brought in favor of this process, the following are opposed to it, viz: that its performance is extremely difficult and that the eye is exposed to great danger during and after the operation.

Now the incision of the cornea—that it may unite by the first intention and without any cicatrix, which would impede the rays of light passing through the cornea, must form the perfect segment of a circle, perfectly regular and smooth at its margins, at a certain distance from the sclerotic coat ; and the aperture must be sufficiently large to permit the easy escapement of the cataract.

Now in the first period of the operation, which is the section of the cornea, and the subsequent one of opening the capsules, the texture of that delicate membrane, the Iris, must be respected. Among the principal dangers attending this operation, is the evacuation of the vitreous Humor. If the Hyaloid membrane is not perfectly healthy, it is apt to be ruptured ; and the Vitreous Humor expelled from the eyes, either before, with, or subsequent to the expulsion of the opaque Lens. There remains after the most favorable case of Extraction, an extensive wound of the cornea ; and the closure of this wound by immediate union and without any protrusion of the Iris, we conceive to be a peculiar desideratum. If the Iris should project into the wound, it complicates the case.

Sometimes violent suppurative inflammation invades the eye after this process ; the entire structure of the organ is broken down, and becomes a disorganized mass.

In less severe cases, the violence of the inflammation, visits that delicate structure called the Iris ; the pupil closes or the cornea becomes opaque.

To excise circularly with a knife, a tissue surrounding a fluid—which fluid is not to be discharged until that section is carried to the extent of a semicircle ; and within this fluid suspended a movable membrane ; which must not be displaced or injured in performing this section—to do this well, is one of the most delicate manipulations on the Human Eye, and characteristic of the movements of a master-hand; few possessiug the manual dexterity required for its successful execution. Unless we have frequent opportunities of operating, we cannot become accustomed to the various difficulties of the process and acquire that confidence in our own power which is so essential to its successful termination. The constant rolling of the organ, the little control which the patient has over it, the mind excited by the dread of the operation : and the convulsive or spasmodic actions of the organ when brought into actual contact with the instruments, are sources of real difficulty, so that the greatest care and judgment are required to bring the case to a successful termination. Undoubtedly Extraction is a process of extreme delicacy, and unless we frequently perform it on the Cadaver, so as to acquire an idea of the resistance of the tissues ; the expectation of succeeding on the living subject, would be as unreasonable, as the attempts would be unjust and absurd. I believe these comprise the principal arguments, in favor of and against Extraction. I shall now take up Depression or Couching, and its modification, Reclination.

The principles on which the process of depression is founded are essentially bad. In this operation we push the opaque Lens directly below the axis of vision, and necessarily rupture a number of the ciliary processes; and produce injurious pressure on the posterior surface of the Iris; as there is really no appreciable space between this delicate membrane and the capsule of the Lens, and consequently no posterior chamber.

It is on this principle that A. Von Graefe, the Berlin Occulist's new operation called Iridectomy, for the cure of Glaucoma is founded, reducing the extreme tension of the Globe, by the evacuation of the acqueous humor, and consequently relieving the pressure of the ball and Retina; leaving more space for the fluid, by excision of the Iris. Another objection to this process is, that the Lens is only partially covered by the vitreous humor; and its elasticity is very apt to cause it to ascend again and impede vision. There is also great danger from pressure on the Retina producing Amaurosis.

I believe these are the views generally entertained by Opthalmic Surgeons with respect to this process.

I shall now take up Reclination. As there are some peculiarities in my manner of performing this operation, which differs from that usually laid down in books, I shall take the liberty to describe it.

We are generally directed by books to introduce the needle, a certain distance from the cornea, above or below the transverse diameter of the eye. Now if we recollect our anatomy, the long ciliary artery comes up on each side, and bifurcates three lines from that membrane, so that we can introduce the needle in the centre of the bifurcation, two lines from the cornea, without interfering with those vessels. The advantage of making the puncture at this point, consists in having easier access to the upper or lower edge of the cataract, so as to incise the capsule fully.

There is one circumstance connected with this process; and on which its chief utility depends, and which I wish to draw attention to, so that others may verify the fact.

I have been operating on the Human Eye for a number of years, and, I have never known it to fail in a single case, no matter how tremulous the Iris was, indicating a dissolution of the vitreous humor. If the following directions are implicitly followed, the cataract will remain reclined.

In the first place introduce the needle two lines from the cornea in the transverse diameter of the eye, with the cutting edges backward and forward: by this means you pass it as it were between the fibres of the sclerotic coat and the ciliary processes, without tearing or lacerating them. As soon as the curved portion is introduced, rotate the needle on its own axis, so as to bring the convex portion of the curve forward, and by that means you pass it between the Iris and the capsule of the Lens, without injuring that delicate and fragile membrane. Now give the cutting edge that direction that it will bear on the capsule of the Lens, so as to incise it freely, then bring the convexity of the curve forward, a little above the transverse diameter of the cataract in front; make the handle of the instrument follow the direction of a line forward, upward and inward; this will compel the cataract to describe a course, backward and outward, making the aperture in the sclerotic coat the fulcrum; this will recline the cataract, making its anterior surface uppermost, and its lower edge forwards, placing it in that portion of the vitreous humor, between the external and inferior recti muscles; turn the needle frequently on its axis to free its point from the cataract; then withdraw it, in the reverse position it was entered, with the convex portion forward until you free it from the Iris; and then turn the cutting edges backward and forward as you withdraw it through the external puncture. At this period of the operation you will have to be extremely cautious, otherwise the Lens may revolve on its axis and pass into the anterior chamber; in that case, the best practice is to extract immediately by a small superior section of the cornea.

I cannot explain the cause of the detention of the cataract at this particular point; it must be that the vitreous humor is more consistent here than elsewhere; but why it should be so, I am at a loss to hazard an opinion. The ultimate success of this process, depends on a free laceration of the chrystaline capsule; so that the humors of the eye may have free access to the cataract, and in this manner be taken up by absorption.

I am well aware that a number of the profession are opposed to this process, under any circumstances; and even go so far as to contend that it is only palliative, and that the Lens is liable at any moment to ascend again and impede vision. It must be freely admitted, that if the Lens is reclined, surrounded with its capsule, it is impossible for it to absorb; but if freed from that membrane, it will gradually disappear. In support of this argument I shall give you the names of Dr. W.

Sommerring, Hey and M. Lisfranc. These gentlemen have dissected eyes after death where reclination had been performed. The Lens had entirely disappeared. On the contrary, Emden, Beer and Hey, who have examined eyes after death, found the Lens reclined with its capsule undisturbed, over which absorption seemed to have no control; proving that, as I mentioned before, depriving the Lens of its capsule is a very important point for the successful termination of this operation.

Since my residence here I have had two cases, of hard capsulo-lenticular cataract, of the ages of 60 and 64 years. One of them, William Legget, resides in this place, and may be seen at any time. His Iris still remains tremulous, showing a dissolved state of the Vitreous Humor. I reclined his cataract, (one eye only being operated on by me) and his sight is perfect with the aid of a cataract glass.

This was one of the most unfavorable cases you could well meet with; for his general health was very much impaired, in consequence of a broken down constitution; the other case, aged 60, had the same diseased state of the Vitreous Humor; but his general health was much better. He also recovered his vision. I had several other cases, but I select these two as being immediately in this neighborhood.

Before leaving this subject I beg leave to say a few words on constitutional treatment. This is all important Get the patient's health up to the highest possible point of perfection you can, before you commence to operate; and this will make all the difference between losing and gaining vision.

Now in reviewing the arguments for and against this modification of Depression, called Reclination, I conceive that a mind untrammeled by prejudice will not cast that odium and stigma upon it, which some authors have done.

I do not for a moment wish to inculcate the principle that this process should supercede Extraction or Solution; by no means; but the principle I contend for is, that there is a certain class of cases, which this process is applicable to, and no other; and I am not aware of any manner, in which a person could exhibit his ignorance of opthalmic surgery more grossly; than by asking what is your favorite mode of operating for cataract; as one class of cases will come under the process of solution or absorption; another under Reclination, and another under Extraction.

I shall simply state a few cases in support of this argument; for instance, persons present themselves advanced in life, with double cataract—(now in those individuals we *generally* meet with hard cataract, I say *generally*, for sometimes, rarely however, they are soft in that class of people)—with a small palpebral fissure, very shallow anterior chambers, so as to endanger wounding the Iris in making the section of the cornea, and a sunken eye; and that may be produced by two causes; 1st, an unusual projection of the supra orbital ridge; 2d by absorption of the adipose tissue. In these cases you cannot denude the globe sufficiently without producing such an amount of pressure as would evacuate the Vitreous Humor and consequently cause the destruction of the organ. Advanced age in itself is also an objection; then the recuperative powers of nature are enfeebled, and consequently the adhesive process would fail and the section of the cornea would not heal by first intention—a broken down constitution, independent of age, would form an analagous ground of objection; adhesions of the pupil; affections of the chest producing cough, difficulty of breathing &c., &c., myosis, or habitual contraction of the pupil; the eyes unsteady and easily thrown into convulsive movements; excessive and insuperable timidity, rendering them unmanageable during and after, the operation; a dissolved state of the Vitreous Humor.

These are all cases in which Extraction or solution would be entirely inapplicable, and out of the question. The only possible chance would be by Reclination.

Permit me to make a few brief remarks to those commencing opthalmic Surgery.

The Human Eye, as we are all well aware, is a minute but very complex organ, and has a representative of every tissue building up the Human fabric. It is as it were, an exquisite but diminutive mirror, wherein we see reflected the phenomena of the disease, and wherein the therapeutical effects of certain remedies are open to our inspection.

Sight is certainly our most precious sense, and indeed we may well say that the Human Eye is God's Telegraph between mind and matter. Through this medium all our other senses are made subordinate to each other. It is also our most precious channel of communication with the external world, and with each other. Man is naturally a social being, consequently blindness is one of the greatest afflictions that which can befal him; and indeed some would prefer to die, rather than, isolated from society, to drag out a miserable existence, shut out from the cheering light of day, and deprived of the pleasure of seeing those whom they love.

Even to the wealthy, the loss of sight is an inexpressible grief and anguish. Wealth may, indeed, somewhat mitigate their sufferings, by enabling them to purchase assistance from others. But when this misfortune visits the poor and needy; the working classes of society, who are depending on their daily labor for support, what a scene of wretchedness and misery is unfolded to their view; their intellect uncultivated perhaps by education; not one ray of hope to illuminate their dark and dreary journey through this cold, unfeeling, and uncharitable world, with scarcely sufficient to nourish and support their emaciated forms. At last, God, in his infinite wisdom, takes them to himself, and they finally sink into a pauper's grave Now this is not by any means an exaggerated, or highly painted picture of the imagination, but one we frequently witness, from the effects of some ill advised, and badly executed operation on the human eye. This should act as a powerful incentive, to admonish and warn us of our imperative duty; which is to avail ourselves of every means in our power, to enlighten and improve our minds on this subject. We surely ought not to attempt to practice this department of our profession, without due and sufficient preparation; capable of doing so much good, if properly studied and understood; so full of danger and peril to the human family, if shrouded with ignorance and recklessness. I would fain hope the responsibility which this view of the subject reveals to us, will, as I have said before, induce every honorable and high-minded man to direct his undivided attention to the various diseases of this complicated, delicate and most precious organ; and to take every advantage, both by dissection and otherwise, to obtain that knowledge and information which will enable him to diminish and mitigate the sufferings of thousands yet unborn; recollecting at the same time, in our most fortunate cases we are only the favored instruments of a supreme and all ruling power who guides our destinies through this world, and is the foundation and source from whence all that is pure and holy emanates.—*Maine Med. & Sur. Reporter..*

